# Culture and expansion of murine proximal airway basal stem cells

**DOI:** 10.1186/s13287-024-03642-2

**Published:** 2024-01-30

**Authors:** Meirong Wu, Xiaojing Zhang, Yanjuan Tu, Wenzhao Cheng, Yiming Zeng

**Affiliations:** 1 Department of Pulmonary and Critical Care Medicine, Second Affiliated Hospital of Fujian Medical University, Quanzhou, Fujian Province People’s Republic of China; 2 Fujian Key Laboratory of Lung Stem Cells, Second Affiliated Hospital of Fujian Medical University, Quanzhou, Fujian Province People’s Republic of China; 3 Jinan Microecological Biomedicine Shandong Laboratory, Jinan, Shandong Province People’s Republic of China; 4 Department of Pathology, Second Affiliated Hospital of Fujian Medical University, Quanzhou, Fujian Province People’s Republic of China

**Keywords:** Proximal airway, Basal cells, Expansion, Self-renew, Differentiation

## Abstract

**Background:**

The stem cell characteristic makes basal cells desirable for ex vivo modeling of airway diseases. However, to date, approaches allowing them extensively in vitro serial expansion and maintaining bona fide stem cell property are still awaiting to be established. This study aims to develop a feeder-free culture system of mouse airway basal stem cells (ABSCs) that sustain their stem cell potential in vitro, providing an experimental basis for further in-depth research and mechanism exploration.

**Methods:**

We used ROCK inhibitor Y-27632-containing 3T3-CM, MEF-CM, and RbEF-CM to determine the proper feeder-free culture system that could maintain in vitro stem cell morphology of mouse ABSCs. Immunocytofluorescence was used to identify the basal cell markers of obtained cells. Serial propagation was carried out to observe whether the stem cell morphology and basal cell markers could be preserved in this cultivation system. Next, we examined the in vitro expansion and self-renewal ability by evaluating population doubling time and colony-forming efficiency. Moreover, the differentiation potential was detected by an in vitro differentiation culture and a 3D tracheosphere assay.

**Results:**

When the mouse ABSCs were cultured using 3T3-CM containing ROCK inhibitor Y-27632 in combination with Matrigel-coated culture dishes, they could stably expand and maintain stem cell-like clones. We confirmed that the obtained clones comprised p63/Krt5 double-positive ABSCs. In continuous passage and maintenance culture, we found that it could be subculture to at least 15 passages in vitro, stably maintaining its stem cell morphology, basal cell markers, and in vitro expansion and self-renewal capabilities. Meanwhile, through in vitro differentiation culture and 3D tracheosphere culture, we found that in addition to maintaining self-renewal, mouse ABSCs could differentiate into other airway epithelial cells such as acetylated tubulin (Act-Tub) + ciliated and MUC5AC + mucus-secreting cells. However, they failed to differentiate into alveoli epithelial cells, including alveolar type I and alveolar type II.

**Conclusion:**

We established an in vitro feeder-free culture system that allows mouse ABSCs to maintain their stem cell characteristics, including self-renewal and airway epithelium differentiation potential, while keeping up in vitro expansion stability.

**Supplementary Information:**

The online version contains supplementary material available at 10.1186/s13287-024-03642-2.

## Background

In the human and mouse proximal airways (trachea and main bronchi), ABSCs are traditionally considered the primary stem cells [1–3] essential for airway tissue homeostasis, with significant self-renewal capacity, as well as the ability to differentiate into luminal airway cells including secretory cells, ciliated cells, mucus-secreting goblet cells, and some smaller cell populations such as lung neuroendocrine cells, tuft cells, and ionocytes [1, 3–11].

As resident stem cells, ABSCs are crucial for airway tissue homeostasis. The stem cell property makes them a desirable ex vivo-producing cell type for airway disease modeling and a principal candidate in stem cell-based regenerative medicine. The in vitro culture models have been simple and powerful tools for exploring ABSC biology [12, 13]. However, a proper cultivation system that allows ABSCs extensive in vitro serial expansion and preserves their stem cell property is still awaiting to be established.

In clinical practice, obtaining normal human airway epithelial cells is often difficult due to the minimal sources and certain ethical restrictions. Since mouse airway tissue is highly conserved with human airway tissue in terms of tissue structure, physiological properties, cellular composition, and biological characteristics [14–16], mouse airway tissue is an excellent experimental platform for simulating the human airway [14]. Construction of the isolation and culture system of ABSCs and studying their stem cell characteristics through establishing a mouse model will significantly promote understanding of the characteristics of human ABSCs and the clinical application of stem cell therapy in lung diseases.

**Fig. 1:**
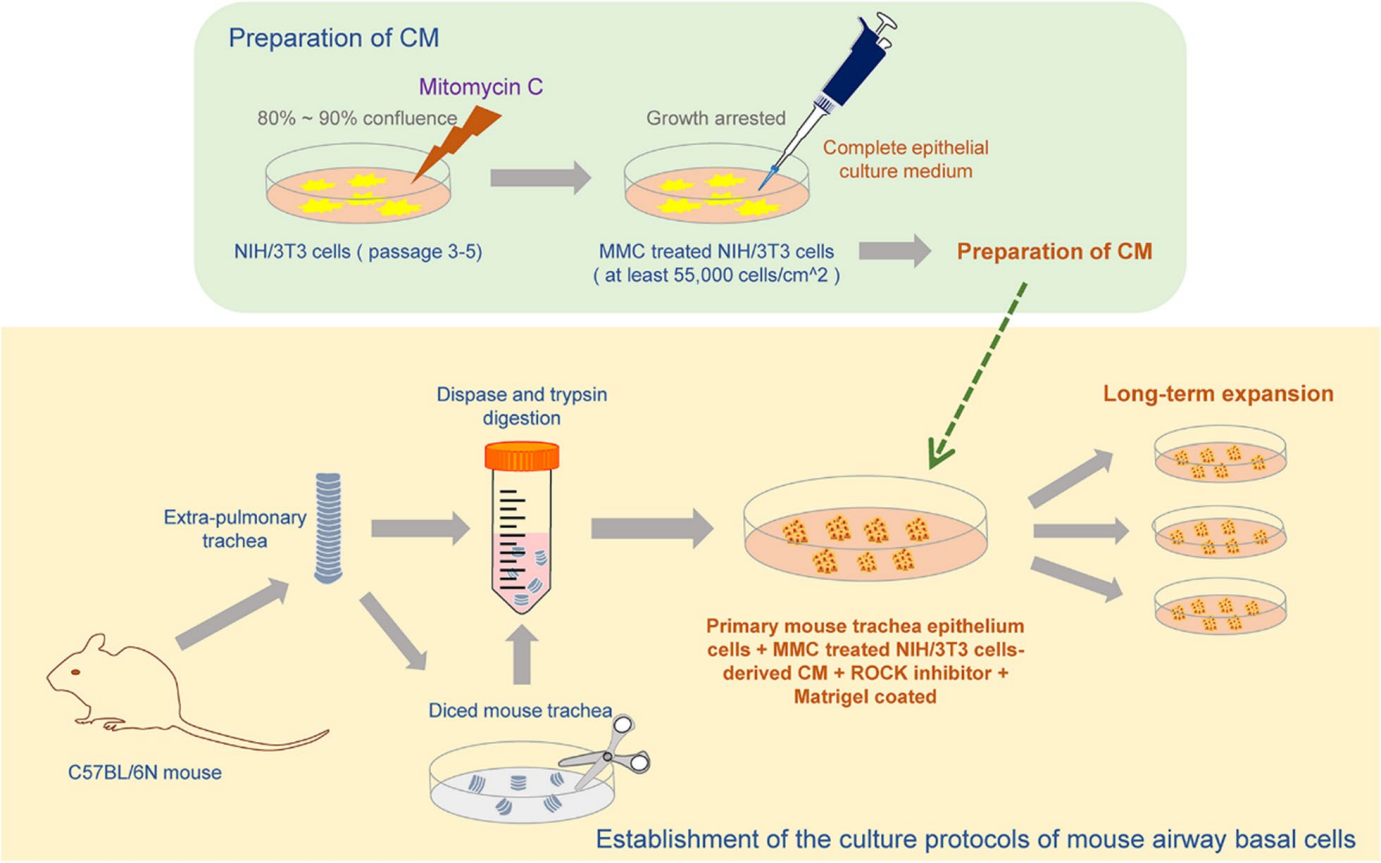
Schematic diagram of the feeder-free culture protocols of mouse ABSCs. Mouse airway tissues are digested into a single-cell suspension, seeded in a cell culture dish pre-coated with Matrigel, and maintained culture of mouse ABSCs using 3T3-CM containing ROCK inhibitor Y-27632

Here, we developed a feeder-free mouse ABSCs culture approach that could stably proliferate in vitro and sustain self-renewal and differentiation potential (Fig. 1), providing an experimental basis for further in-depth mechanism exploration in the future and offer new ideas for the treatment of lung tissue stem cells in pulmonary disease.

## Methods

### Feeder layers generation and preparation of conditioned medium (CM)

All experimental animal procedures adhered to Fujian Medical University’s Policy on the Care and Use of Laboratory Animals and were approved by the Ethics Committee of the Second Affiliated Hospital of Fujian Medical University (Approval Number 2022-454).

NIH/3T3 mouse embryonic fibroblasts were purchased from the Guangzhou Jennio Biotech Co., Ltd. and cultured in Dulbecco’s modified Eagle’s medium (DMEM; Gibco) supplemented with 100 U/mL penicillin, 100 μg/mL streptomycin (Gibco), and 9% newborn calf serum (NBCS; Gibco). Primary mouse embryonic fibroblasts (MEFs) were isolated from CF-1 mice at 12.5 days of gestation, and primary rabbit embryonic fibroblasts (RbEFs) were isolated from 20 to 22 days pregnant New Zealand rabbits according to the previously described method [17]. MEFs and RbEFs were cultured in DMEM containing 10% fetal bovine serum (FBS; Gibco), 100 U/mL penicillin, and 100 μg/mL streptomycin. Cells were cultured at 37°C in a 5% CO_2_ incubator with three medium changes per week. For feeder layers generation or preparation of CM, NIH/3T3 cells were mitotically inactivated by treatment with a final concentration of 4 μg/mL mitomycin C (MMC; Selleck), and MEFs and RbEFs cells were treated with 10 μg/mL MMC in a culture medium for 2 h. Cells were trypsinized and re-seeded at a density of at least 20,000 cells/cm^2^ in the growth medium for feeder layers generation or at least 55,000 cells/cm^2^ to prepare CM. When used as feeder layers, mouse proximal airway epithelial cells (MPAECs) suspended in an MPAEC culture medium were added the following day. When preparing CM, MPAEC basic medium was added the following day, and the CM was collected daily subsequently. MPAEC basic medium consists of serum-containing DMEM (500-mL DMEM plus 50-mL FBS, 100 U/mL penicillin, and 100 μg/mL streptomycin) and Ham's F12 (Gibco) at a 3:1 ratio supplemented with 1 × gentamicin/amphotericin B solution (Gibco). MPAEC culture medium is defined as MPAEC basic medium supplemented with additive to achieve a final containing a concentration of 25 ng/mL hydrocortisone (MCE), 0.125 ng/mL recombinant mouse epidermal growth factor (Gibco), 4 ng/mL basic FGF (R&D Systems), 0.1-nM cholera toxin (MCE), 5 mg/mL insulin (MCE), 5-mM ROCK inhibitor Y-27632 (Selleck), and 1000 U/mL leukemia inhibitor factor (LIF; Millipore). The same additive was added to CM before feeding cells on Matrigel (BD Biosciences). Mitotically inactivated feeder cells can be used for up to 10 days.

### MPAEC isolation and culture

Referring to the previously described method, murine extra-pulmonary tracheas were obtained from wild-type C57BL/6N mice and transported on ice in an αMEM medium containing penicillin–streptomycin and amphotericin B [18]. Extra-pulmonary tracheas were digested to single-cell suspensions in serum-free RPMI medium (Gibco) containing 16 U/mL dispase (Corning) for 20 min and following 0.1% trypsin–EDTA (0.25% trypsin–EDTA diluted in RPMI medium) (Gibco) in a water bath at 37 °C for 30 min with regular agitation. After isolation, the mouse trachea tissue blocks or digested single-cell suspensions were plated on preprepared feeder layers in MPAEC culture medium or seeded on pre-coated Matrigel in CM supplemented with additive. MPAECs were cultured at 37 °C and 5% CO_2_ with 3–4 medium refreshes weekly. MPAECs were passaged by dissociation using TrypLE Select (Gibco) at 75–85% confluence.

### Evaluation of population doubling time (PDT)

For the evaluation of PDT, mouse proximal ABSCs were counted when subculture and incubated in Matrigel pre-coated 10-cm cell dishes (Coming) at 37 °C with 5% CO_2_ 10–14 days until the clones were formed and reached 70–80% confluent. The PDT was calculated using the formula: PDT = Δ*t* × Lg2/ [Lg (cells harvested/cells seeded)]. Δ*t*: duration of culture (hrs).

### Evaluation of colony-forming efficiency (CFE)

To analyze colony-forming capacity, mouse proximal ABSCs were seeded onto Matrigel pre-coated 6-well plates (Corning) at 250 cells per well for 10–14 days until the clones were formed and reached 75–85% confluent. Plates were fixed for 10 min with 4% (*w*/*v*) paraformaldehyde (PFA; Boster) and stained using 1% crystal violet solution (Sigma). Plates were washed extensively in water and allowed to dry at room temperature overnight. Colonies were counted using a brightfield microscope. Total colonies were counted in triplicate wells per sample per passage, and CFE was calculated by the number of colonies formed/number of cells seeded multiplied by 100%.

### In vitro differentiation cultures

Mouse proximal ABSCs were seeded on Matrigel-coated 6-well plates in the submerged culture at 1000 cells per well and allowed to spontaneously differentiate in differentiation medium (DM) composed of 80% knock-out DMEM (Gibco), 20% FBS, 1% nonessential amino acids (NEAA; Gibco), 1-mM L-glutamine (Gibco), 100 U/mL penicillin, and 100 μg/mL streptomycin for 16 days [19]. The medium was changed every other day.

### Immunocytofluorescence (ICF) in cell cultures

Cells in expansion culture or after in vitro differentiation culture were rinsed with Dulbecco’s phosphate-buffered saline (DPBS; Biological Industries) followed by fixation with 4% (*w*/*v*) PFA for 10 min at room temperature and washed three times with DPBS. Cells were stored at 4 °C in DPBS until staining. Cells were permeabilized for 10 min in DPBS containing 0.2% (*v*/*v*) Triton X-100 (Sigma-Aldrich) and washed thrice with DPBS. Autofluorescence quench was performed by incubating in 1X TrueBlack® lipofuscin autofluorescence quencher (Biotium) diluted in 70% ethanol for 10 min and washed three times with DPBS. After TrueBlack® treatment, buffers containing detergent cannot be used. Non-specific binding sites were blocked in PBS containing 5% (*w*/*v*) bovine serum albumin (BSA; Sigma-Aldrich), 5% (*v*/*v*) donkey serum (Solarbio), 5% (*v*/*v*) goat serum (Beyotime), and 0.3 M glycine (Solarbio) for 1.5 h at room temperature, followed by incubation with the appropriate primary antibodies in blocking buffer at 4 °C overnight: p63 (1:100; R&D), Cytokeratin 5 (1:200; Invitrogen); Mucin 5AC (1:250; Bioss); Acetylated Tubulin (Act-Tub) (1:200; Millipore); Prosurfactant Protein C (pro-SPC) (1:1000; Millipore); and Podoplanin (1:250; Abcam). The next day, cells were washed six times with DPBS and incubated with secondary antibodies (Alexa Fluor 488, or 546, 1:1000; Invitrogen) in a blocking buffer for 1 h at room temperature. After five washing steps with DPBS, the cells were counterstained with 4′,6-diamidino-2-phenylindole (DAPI; Beyotime) at 1:2000. Cells were washed three times with DPBS and kept in DPBS for imaging with a Leica inverted fluorescence microscope.

### Three-dimensional tracheosphere assay

To generate differentiated 3D airway cultures, mouse proximal ABSCs were seeded on ultra-low attachment 6-well plates (Corning) at 20,000 cells per well and allowed to differentiate spontaneously. Cells were fed by DM or CM supplemented with additive except for LIF (NL-CM) for 21 days. Every 2–3 days, the cultured tracheospheres were collected and centrifuged at 200 g for 3 min for medium changes. On day 21, tracheospheres were collected and fixed by resuspension in 4% PFA for 30 min and washed with DPBS, followed by paraffin embedding and histology processing [20].

### Histology and immunohistofluorescence (IHF)

After fixation, tracheospheres were embedded in paraffin and sectioned at 5 μm with a Leica microtome. Hematoxylin and eosin (H and E) staining was performed. For IHF, slides were dewaxed, rehydrated, and subjected to antigen retrieval in 1 × Citrate Buffer pH 6.0 (Abcam) diluted in water at 95–100 °C for 15 min and 20 μg/mL Proteinase K (Beyotime) diluted in TE buffer (50-mM Tris, 1-mM EDTA, pH 8.0) at 37 °C for 10 min, followed by permeabilized for 30 min in DPBS containing 1% (v/v) Triton X-100. Autofluorescence quenches, non-specific binding sites blocking, primary antibodies incubation, second antibodies incubation, and counterstain of DAPI were performed in the conditions described in the above section “ICF in cell cultures.” Sections were mounted with ProLong™ Diamond Antifade Mountant (Molecular Probes), and images from IHF slides were acquired using a Leica inverted fluorescence microscope.

### Statistical analysis

Statistical analyses were performed, and statistical graphs were plotted with GraphPad Prism, version 8.0 (GraphPad Software, San Diego, CA, USA). All data are presented as the means ± standard deviations (SD). The comparison between two groups was determined using an independent Student’s t-test and between more than two groups using a one-way analysis of variance (ANOVA) followed by the Tukey multiple comparison test. *P* value < 0.05 was designated statistically significant.

## Results

### 3T3-CM containing ROCK inhibitor Y-27632 well preserves mouse ABSCs

**Fig. 2:**
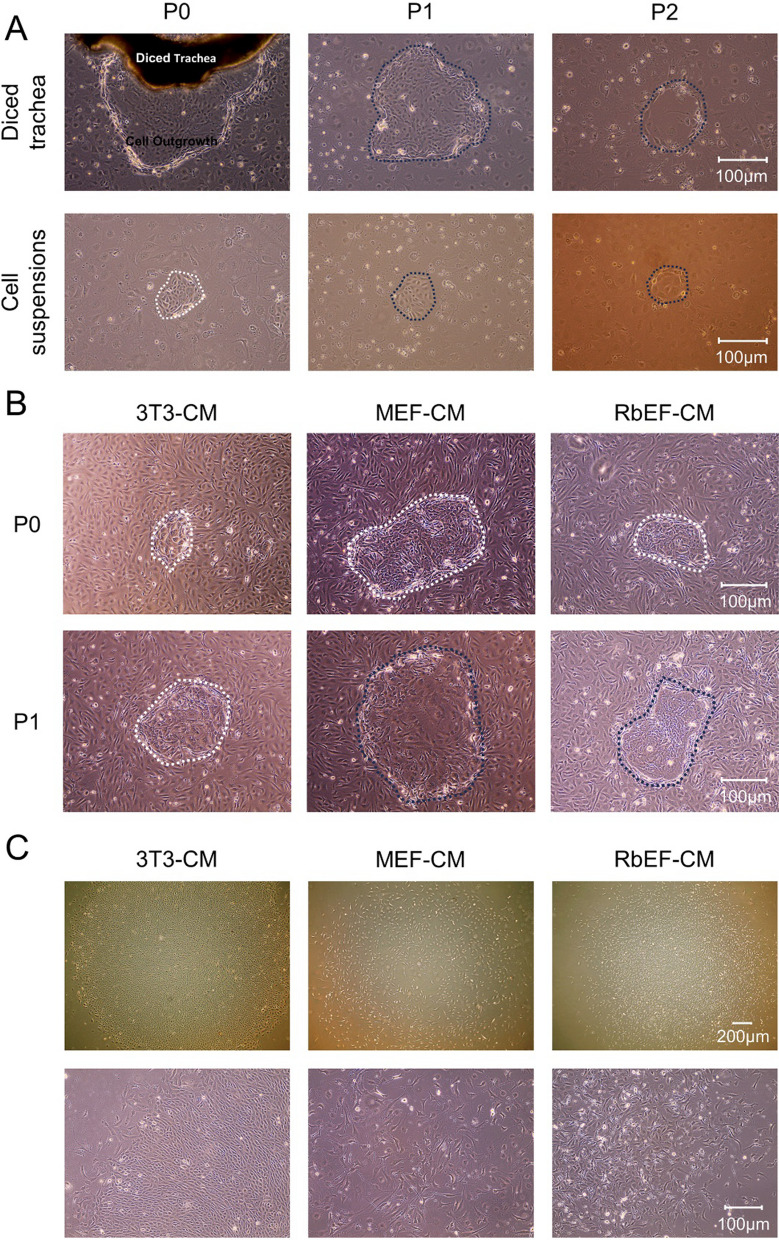
Morphological manifestations of mouse ABSCs during culture. **A** Brightfield images showing the cell morphology of P0, P1, and P2 airway epithelial cells derived from diced tracheal tissue or cell suspensions produced by dispase and trypsin digestion co-cultured with pre-seeded MMC-treated NIH/3T3 cells in MPAEC culture medium containing Y-27632. White dotted lines indicate the well-defined stem cell-like clones, while blue dotted lines indicate that cells in the clones were undergoing differentiation. **B** Well-defined stem cell-like clones were observed in P0 during culture with 3T3-CM, MEF-CM, or RbEF-CM containing Y-27632. In the P1, when cultured with 3T3-CM, cells in the clones kept a typical cobblestone shape and expanded with minimal cell–cell contact, while in MEF-CM and RbEF-CM culture, some cells in the clones began to differentiate. White dotted lines indicate the well-defined stem cell-like clones, while blue dotted lines indicate that cells in the clones were undergoing differentiation. **C** In the P5, well-defined stem cell-like clones were maintained only in the 3T3-CM containing Y-27632 culture system, whereas when cultured with MEF-CM or RbEF-CM, cells within the clones began to undergo differentiation

NIH/3T3 cells exhibited a typical fusiform morphology during culture (Additional file 1: Fig. S1A). The isolated mouse trachea tissue blocks or digested single-cell suspension were inoculated into pre-seeded mitotically inactivated NIH/3T3 cells. The MPAEC culture medium containing ROCK inhibitor Y-27632 was used for culture. As shown in Fig. 2A, epithelial outgrowths with a clear border are visible 7 days after diced tracheal tissue inoculation. Most of the cells were cobblestone-shaped; a few seemed large and flat (P0, Fig. 2A, top left). Meanwhile, after 3 days of inoculating the single-cell suspension, well-defined stem cell-like colonies of smaller epithelial cells were formed and expanded with minimal cell–cell contact (P0, Fig. 2A, bottom left). However, cells became larger and flatter, and the cytoplasm became lighter over passage (P1 and P2, Fig. 2A, middle and right). These indicate that mouse ABSCs could not be stably expanded in this culture system, and a more optimized is necessary to explore.

Afterward, we used NIH/3T3-derived CM to seek a more optimized culture system, and the cell dishes were pre-coated with Matrigel. As representative feeder cells, primary MEFs and RbEFs-derived CM are also used in the following experiments. As shown in Additional file 1: Fig. S1B and C, the miscellaneous cells gradually disappeared with culturing. By the P3, the cell morphology was consistent, shaped in spindles with protruded pseudopodia, and relatively pure MEFs and RbEFs were obtained. By the P5, the pseudopodia of the cells increased, and the stereoscopic sense was weakened, suggesting a senescence state. Therefore, we used the P3 MEFs and RbEFs in the following study.

As shown in Fig. 2B, regardless of culturing by 3T3-CM, MEF-CM, or RbEF-CM, the P0 mouse airway epithelial cells formed stem cell-like clones with clear boundaries. Cells in the clones were cubic or cobblestone-like and formed tight junctions. In contrast, the cells outside clones were spindled- or cord-shaped, which might consist of other epithelial cells and pulmonary interstitial cells, except for ABSCs. After passing to P1, stem cell-like clones remained in the culture system of 3T3-CM containing Y-27632. By contrast, in the MEF-CM and RbEF-CM culture systems, some cells in the clones became larger and flatter, and the cytoplasm thinner and sparser, suggesting that it underwent differentiation. Cells outside the clone kept spindling- or cord-shaped. Considering that cells outside the clones might affect ABSC self-renewal capacity maintenance, TrypLE Select was used to digest at room temperature for 1–2 min and gently tapped to attempt to remove most spindle cells outside the clone (Additional file 1: Fig. S2). The maintenance culture was continued. As shown in Fig. 2C, until P5, only the 3T3-CM containing Y-27632 culture system amplified the well-defined stem cell-like clones, with cells in which preserving cuboidal morphology expanded with minimal cell–cell contact. In comparison, cells in the MEF-CM or RbEF-CM culture systems could not maintain this kind of small stem cell-like colonies, and their expansion was limited with passage. Thus, the above results suggested that only the 3T3-CM containing Y-27632 culture system well-preserved stem cell morphology of mouse ABSCs until at least P5.

### Identification and maintenance culture of mouse ABSCs

**Fig. 3:**
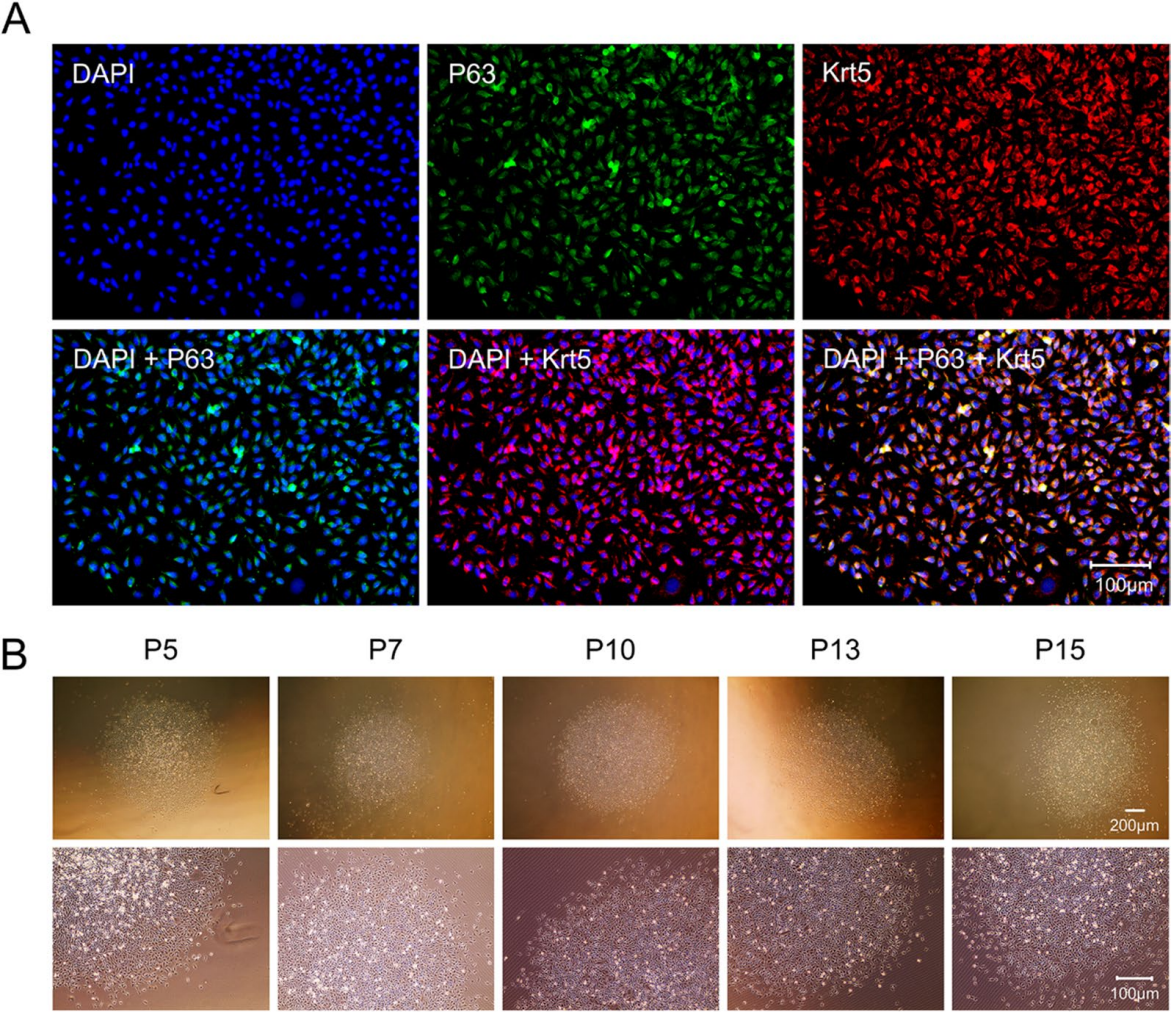
Identification and maintenance culture of mouse ABSCs. **A** Characterization of the cells in colonies. Most were p63/Krt5 double-positive cells (scale bar, 100 μm). **B** Morphological manifestations of mouse ABSCs during maintenance culture. Brightfield images show that mouse ABSCs can maintain the formation of stem cell colonies during passage, and cells within the clones are in a cuboidal or cobblestone shape and retain cell–cell contact

**Fig. 4:**
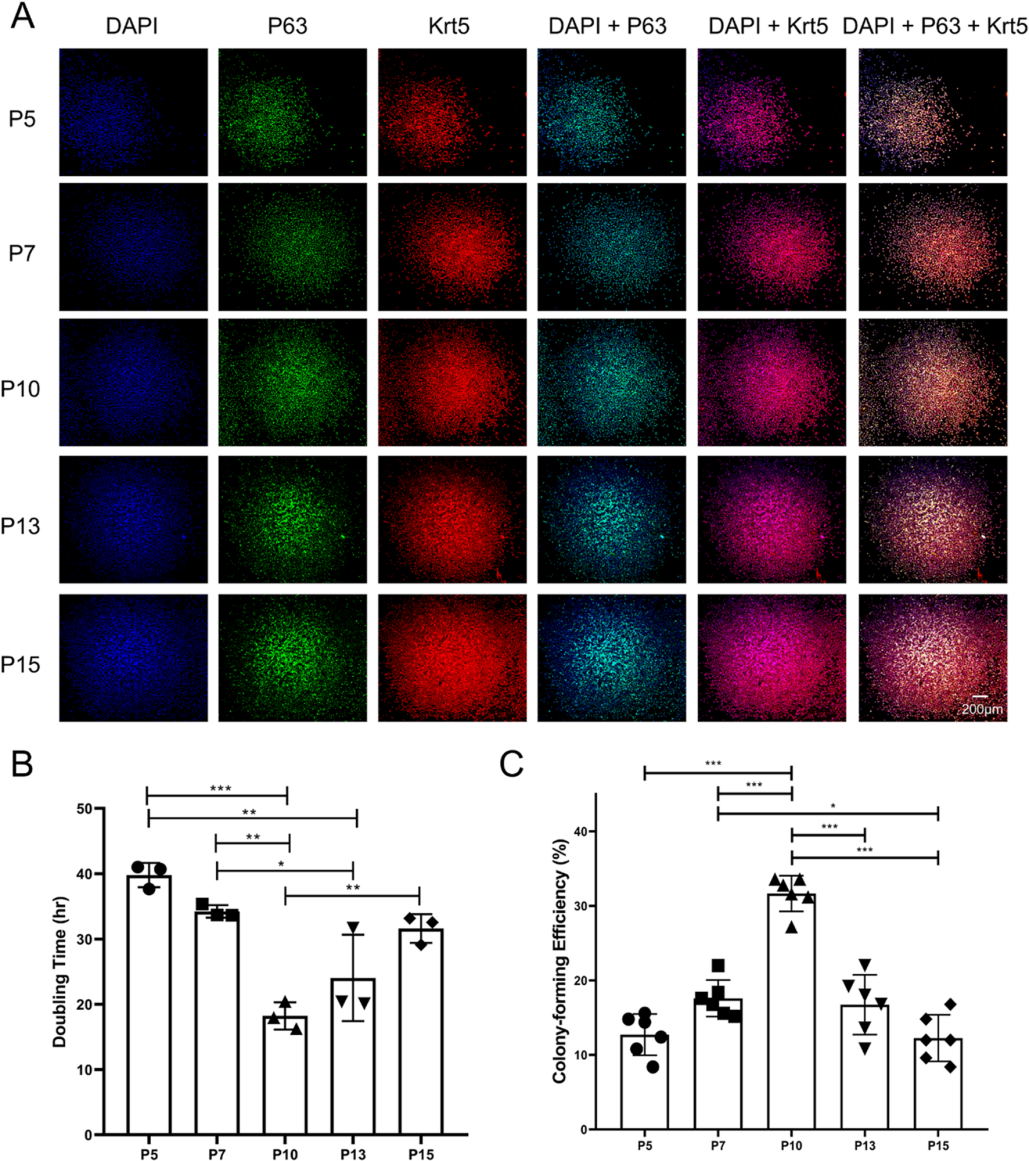
Characterization of the cells during maintenance culture (**A**) and in vitro expansion ability (**B**) and self-renewal capacity (**C**) of mouse ABSCs. **A** During the subculture process, cells in the colonies are p63/Krt5 double-positive cells (scale bar, 200 μm). **B, C** Comparisons of the PDT (**B**, *n* = 3) and CFE (**C**, *n* = 6) of mouse ABSCs at passage 5, 7, 10, 13, and 15. Mouse ABSCs maintain stable in vitro expansion and self-renewal capacity, and the cell cultures from P10 demonstrated the shortest doubling time and the highest colony-forming capacity. The comparison between groups was determined using one-way analysis of variance (ANOVA) followed by the Tukey multiple comparison test. *P < 0.05. **P < 0.01. ***P < 0.001

ICF was used to characterize cells in the obtained clones. As shown in Fig. 3A, nearly all cells (97.37 ± 1.83%, *n* = 3) were p63 positive and co-localized with Krt5, which verified that the clones were composed of p63+/Krt5+ double-positive mouse ABSCs. The maintenance culture was continuously performed. As shown in Fig. 3B, the mouse ABSCs could continuously subculture to at least 15 passages (191 days of continuous culture, equivalent to approximately 130–140 doublings, *n* = 3) and lead to forming bona fide stem cell-like colonies that maintained cell–cell contact, and whose morphology did not change during the culture process. At the same time, it was observed that cells in the clones were positive for p63 and co-localized with Krt5 in the 5, 7, 10, 13, and 15 passages of mouse ABSCs (Fig. 4A). These findings confirmed that mouse ABSCs could continuously subculture to at least 15 passages and maintain their morphology and basal cell markers in the culture system of 3T3-CM containing Y-27632 combined with Matrigel pre-coating dishes.

### In vitro expansion ability and self-renewal capacity of mouse ABSCs

Next, we examined the in vitro expansion and self-renewal capacity by calculating PDT and CFE. As shown in Fig. 4B, it was found that in the 5, 7, 10, 13, and 15 passages, from P5 to P10, the mouse ABSCs maintained a stable in vitro expansion. The PDT decreased gradually, and the PDT at P10 was the shortest. After P10, the PDT of the mouse ABSCs gradually increased with the subculture from P10 to P15 (*P* < 0.05). As shown in Fig. 4C, in the 5, 7, 10, 13, and 15 passages, from P5 to P10, the mouse ABSCs retained a stable self-renewal ability. The CFE gradually increased, and the colony formation ability of P10 was the strongest. After P10, the colony formation ability of mouse ABSCs gradually decreased with the passage from P10 to P15 (P < 0.05). These data suggested that the mouse ABSCs could be preserved to at least P15 in the 3T3-CM containing Y-27632 culture system and maintain the comparable ability of in vitro expansion and self-renewal to early passages. Among them, the P10 ABSCs had the strongest in vitro expansion and self-renewal capacity.

### In vitro differentiation potential of mouse ABSCs

As self-renew and differentiation capacities are indispensable stem cell properties that make ABSCs ideal for in vitro airway disease modeling, we subsequently detected the differentiation potential of mouse ABSCs using in vitro differentiation culture and 3D tracheosphere assay.

**Fig. 5:**
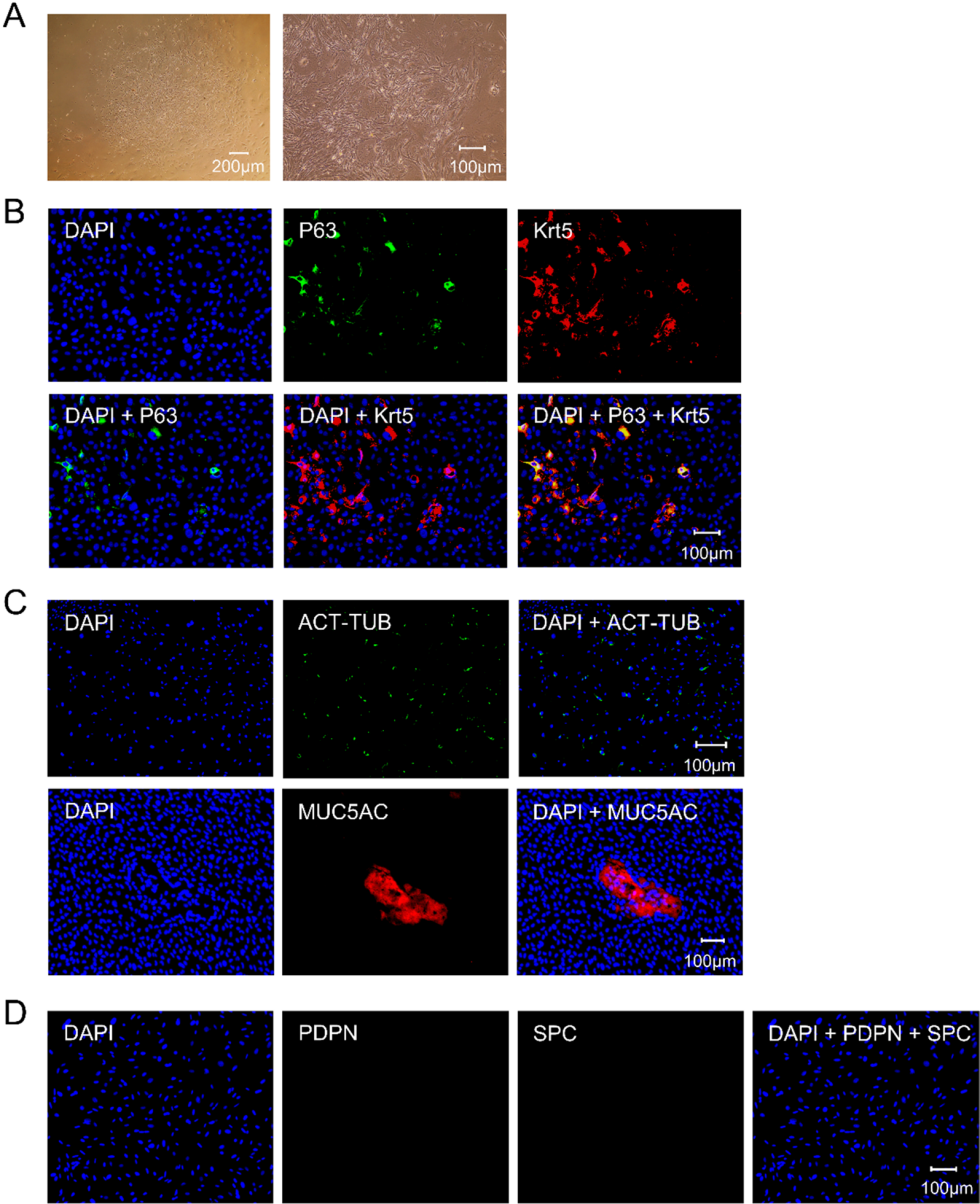
In vitro differentiation culture of mouse ABSCs. **A** Morphological manifestations of cells during in vitro differentiation culture. **B–D** The expression of p63/Krt5 (**B**), Act-Tub (**C**), MUC5AC (**C**), PDPN (**D**), and SPC (**D**) in vitro differentiation culture. During in vitro differentiation culture, mouse ABSCs could maintain the self-renewal of p63/Krt5 double-positive cells (**B**), differentiate into both Act-Tub-positive ciliated cells and MUC5AC-positive mucus-secreting cells (**C**), and not differentiate into both PDPN-positive alveolar type I cells and SPC-positive alveolar type II cells (**E**) (scale bar, 100 μm)

As depicted in Fig. 5A, during the in vitro differentiation culture procedure, the ABSCs underwent differentiation, gradually getting larger and flattening, morphology resembling a spindle, and cytoplasm thinner and sparser. After 16 days of in vitro differentiation culture, the expression of epithelial markers was detected using the ICF method. As shown in Fig. 5B–D, during the in vitro differentiation culture, we observed that mouse ABSCs could maintain self-renewal (p63+/Krt5+; 35.29 ± 4.71%, Fig. 5B). However, compared to continuous culture, the nuclear staining of p63 in mouse ABSCs during in vitro differentiation culture has decreased, with some expression in the cytoplasm. Meanwhile, mouse ABSCs could differentiate into airway pseudostratified epithelial cells containing both ciliated (Act-Tub; 36.62 ± 3.73%, Fig. 5C) and mucosecretory cells (MUC5AC; 10.74 ± 3.33%, Fig. 5C). However, they failed to differentiate into alveoli epithelial cells, including alveolar type I (PDPN; Fig. 5D) and alveolar type II (SPC; Fig. 5D) cells.

**Fig. 6:**
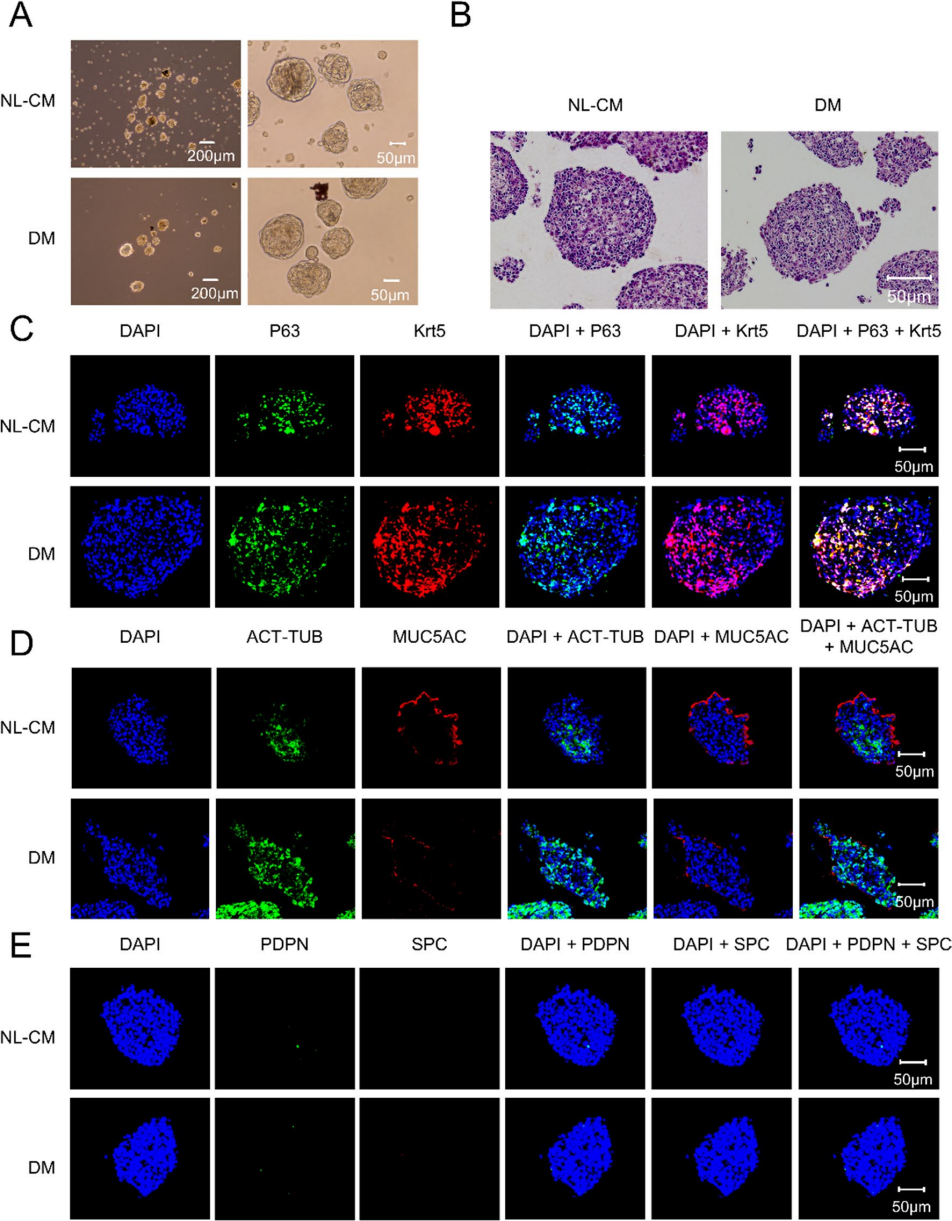
Three-dimensional tracheospheres culture of mouse ABSCs. **A** Brightfield images shows formation of tracheospheres. **B** H and E staining of tracheospheres (scale bar, 50 μm). **C-E.** Using IHF assay to detect the p63/Krt5 (**C**), Act-Tub (**D**), MUC5AC (**D**), PDPN (**E**), and SPC (**E**) expression in tracheospheres generated in 3D culture. During 3D tracheosphere culture, mouse ABSCs could maintain the self-renewal of p63/Krt5 double-positive cells (**C**), differentiate into both Act-Tub-positive ciliated cells and MUC5AC-positive mucus-secreting cells (**D**), and not differentiate into both PDPN-positive alveolar type I cells and SPC-positive alveolar type II cells (**E**) (scale bar, 50 μm)

Moreover, differentiated 3D airway cultures of mouse ABSCs were generated by tracheosphere assay. As shown in Fig. 6A, tracheospheres could be formed in both NL-CM and DM. The H and E analysis indicated that the tracheospheres tissue remained intact, and the nucleus was discernible (Fig. 6B). Meanwhile, IHF was used to detect the expression of lung epithelial markers in tracheospheres formed by 3D culture. Similar to the result of in vitro differentiation culture, we found that cells cultured in 3D tracheosphere assay could stably maintain self-renewal (p63 + /Krt5 + ; NL-CM: 59.38 ± 3.28%; DM: 55.37 ± 3.30%; Fig. 5B), and formed a pseudostratified epithelium containing both ciliated (Act-Tub; NL-CM: 38.24 ± 2.70%; DM: 57.70 ± 3.85%; Fig. 6D) and mucosecretory cells (MUC5AC; NL-CM: 28.54 ± 2.03%; DM: 6.23 ± 1.50%; Fig. 6D). In contrast with the proximal airway epithelial cell markers investigated, ABSCs in 3D tracheosphere cultures did not induce expression of alveoli epithelial cell markers (PDPN and SPC; Fig. 6E). Moreover, compared with tracheospheres culture by NL-CM, a higher proportion of Act-Tub + ciliated cells (*P* < 0.05) and a lower proportion of MUC5AC + mucus-secreting cells (*P* < 0.01) were observed in tracheospheres culture by DM.

The above results from in vitro differentiation culture and 3D tracheosphere assay indicated the maintenance of self-renew and proximal airway epithelial differentiation potential of ABSCs.

## Discussion

The stem cell property makes ABSCs ideal for modeling airway disease in vitro and effectively remodeling airway epithelium based on stem cell regenerative therapies [4, 14]. The in vitro culture model of ABSCs is the simplest and most effective tool for researchers to explore their biological properties. Numerous efforts have been made for in vitro long-term expansion of ABSCs [12, 13, 18]. Recently, Butler et al. co-cultured ABSCs with mitotically inactivated mouse embryonic fibroblast feeder cells 3T3-J2 in a medium containing Y-27632 [12, 18]. Functional ABSCs could be efficiently and long term expanded in this culture method while maintaining airway tissue-specific differentiation capacity and expressing typical markers. However, adding mouse feeder cells to this culture system may interfere with signal transduction pathways related to ABSC proliferation and face challenges in the regulatory requirements for cell therapy product manufacture. Therefore, it is necessary to develop a more stable feeder-free culture system for ABSCs.

It is difficult to obtain human airway epithelial cells in clinical practice, and specific ethical restrictions exist. Since mouse and human airway tissue are highly conserved in tissue structure and cellular composition [14–16], mouse airway tissue is an excellent experimental platform for simulating the human airway [14]. Much of our current understanding of the human airway epithelium is also based on studies on mouse airway tissue.

The conditional reprogramming (CR) culture system referred to the co-culture of mitotically inactivated NIH/3T3 mouse fibroblast feeder layer and digested primary normal or disease-derived cells in the presence of ROCK inhibitor Y-27632, in which the primary cells could acquire stem cell-like characteristics and maintain specific differentiation ability [21, 22]. The NIH/3T3 feeder layer and Y-27632 are critical for CR culture. Treatments for mitotic inactivation of NIH/3T3 feeder layers include irradiation with appropriate doses of radiation or treatment with 2–4 mg/mL MMC for 1–3 h [23]. Mitotically inactivated NIH/3T3 could be used as a feeder layer for co-culture or cultured using NIH/3T3-derived CM containing growth factors well secreted with analogous culture efficiency [24]. CR culture system has significant advantages in cell doubling numbers and maintaining a normal genetic background [25]. Together with organoid culture, the CR culture system is listed as one of the two key new technologies in precision oncology by the National Cancer Institute [22, 26], showing great promise in various biomedical fields such as disease modeling, regenerative medicine, and drug evaluation.

In this study, we construct an improved feeder-free CR culture system of mouse ABSCs that could stably expand and maintain stem cell morphology (Fig. 1). We first attempted to co-culture mouse ABSCs with mitotically inactivated NIH/3T3 cells in a medium containing the ROCK inhibitor Y-27632. However, the mouse ABSCs could not expand in this culture system, which suggested that the CR culture method might not be directly applied to the culture of mouse ABSCs, and it needs to be further improved and optimized. NIH/3T3, MEFs, and RbEFs are representative feeder cells [17, 27–29]. Therefore, 3T3-CM, MEF-CM, and RbEF-CM were subsequently used for further exploration. After exploring and optimizing the culture conditions, it was found that only the 3T3-CM containing Y-27632 culture system amplified the well-defined stem cell-like clones, and cells in the clones preserved cuboidal morphology expanded with minimal cell–cell contact until the P5. In contrast, cells in the MEF-CM or RbEF-CM culture systems underwent differentiation. These might be due to the different cytokines secreted by different types of embryonic fibroblasts, which could be further explored in the future studies [30–32].

Previously, Mou et al. extended a dual SMAD-inhibited in vitro culture system without feeder cells, in which ABSCs could expand to ~ 60–80 doublings and maintain functional airway tissue differentiation ability [13]. Compared to this study, the mouse ABSCs in our established cultivation system could expand to approximately 130–140 doublings. The mouse ABSCs maintain their stem cell characteristics in the maintenance culture, including self-renewal and airway epithelium differentiation potential, while maintaining the in vitro expansion stability. As somatic stem cells, ABSCs could only differentiate into airway epithelium cells, not alveoli epithelial cells. Meanwhile, we found that the nuclear localization of p63 in mouse ABSCs has decreased with more expression in the cytoplasm during in vitro differentiation culture compared to continuous culture. Consistent with the previous research results, these may indicate undergoing a differentiation process [33]. Thus, this study established a feeder-free mouse ABSCs culture system combining 3T3-CM containing the ROCK inhibitor Y-27632 with pre-coating cell culture dishes using Matrigel.

This system uses a CM combined with Matrigel pre-coating to culture mouse ABSCs, avoiding the need for continuous feeder cell culture, continuous mitotic inactivation of cells, and the potential feeder cell contamination risks from co-culturing. This culture system has the following advantages: (1) There is no mixing of exogenous embryonic fibroblasts in the culture system; (2) there is no need for trypsin differential digestion during the subculture process; and (3) the feeder cell-derived CM required in the culture process can be prepared in advance, stored in an ultra-low-temperature freezer at -80°C, and used within 1 month. Although it is necessary to realize that the processing of CM may affect the consistency of cultivation when repeated, such influence could be reduced by improving researcher proficiency and consistency of seeding cell density. This study will lay the foundation for further research on the biological characteristics of ABSCs and provide new ideas for the future application of ABSCs in treating lung diseases.

## Conclusions

This study established an in vitro culture system of mouse ABSCs combining 3T3-CM containing ROCK inhibitor Y-27632 and pre-coating cell culture dishes with Matrigel for the first time (Fig. 1). The mouse ABSCs in this culture system could maintain their stem cell characteristics, including self-renewal and airway epithelium differentiation potential, while maintaining the in vitro expansion stability.

## Fundings

This work was supported by National Natural Science Foundation of China (Grant No. 82070021); Quanzhou High-level Talents Major Project (Grant No. 2020C001R); Doctoral Miaopu Project of the Second Affiliated Hospital of Fujian Medical University (Grant number: BS202306); and the Startup Fund for Scientific Research of Fujian Medical University, China (Grant Nos. 2019QH2040 and 2022QH1107).

### Supplementary Information

**Additional file 1:** Supplementary figures S1 and S2.

## Data Availability

The datasets used and/or analyzed during the current study are available from the corresponding author on reasonable request.

## Abbreviations

ABSCs: Mouse airway basal stem cells
CM: Conditioned medium
DMEM: Dulbecco’s modified Eagle’s medium
NBCS: Newborn calf serum
MEFs: Mouse embryonic fibroblasts
RbEFs: Rabbit embryonic fibroblasts
FBS: Fetal bovine serum
MMC: Mitomycin C
MPAECs: Mouse proximal airway epithelial cells
LIF: Leukemia inhibitor factor
PDT: Population doubling time
CFE: Colony-forming efficiency
PFA: Paraformaldehyde
DM: Differentiation medium
NEAA: Nonessential amino acids
ICF: Immunocytofluorescence
DPBS: Dulbecco’s phosphate-buffered saline
DAPI: 4′,6-Diamidino-2-phenylindole
NL-CM: CM supplemented with additive except for LIF
IHF: Immunohistofluorescence
H and E: Hematoxylin and eosin
SD: Standard deviations
ANOVA: One-way analysis of variance
CR: Conditional reprogramming
