# Supplementary material for: Culture and expansion of murine proximal airway basal stem cells
Source: Stem Cell Res Ther. 2024 Jan 30;15:26. doi: 10.1186/s13287-024-03642-2 (PMC10826159; doi:10.1186/s13287-024-03642-2)
Supplement: Supplementary file 1 — Additional file 1: Supplementary figures S1 and S2. [file 13287_2024_3642_MOESM1_ESM.docx]

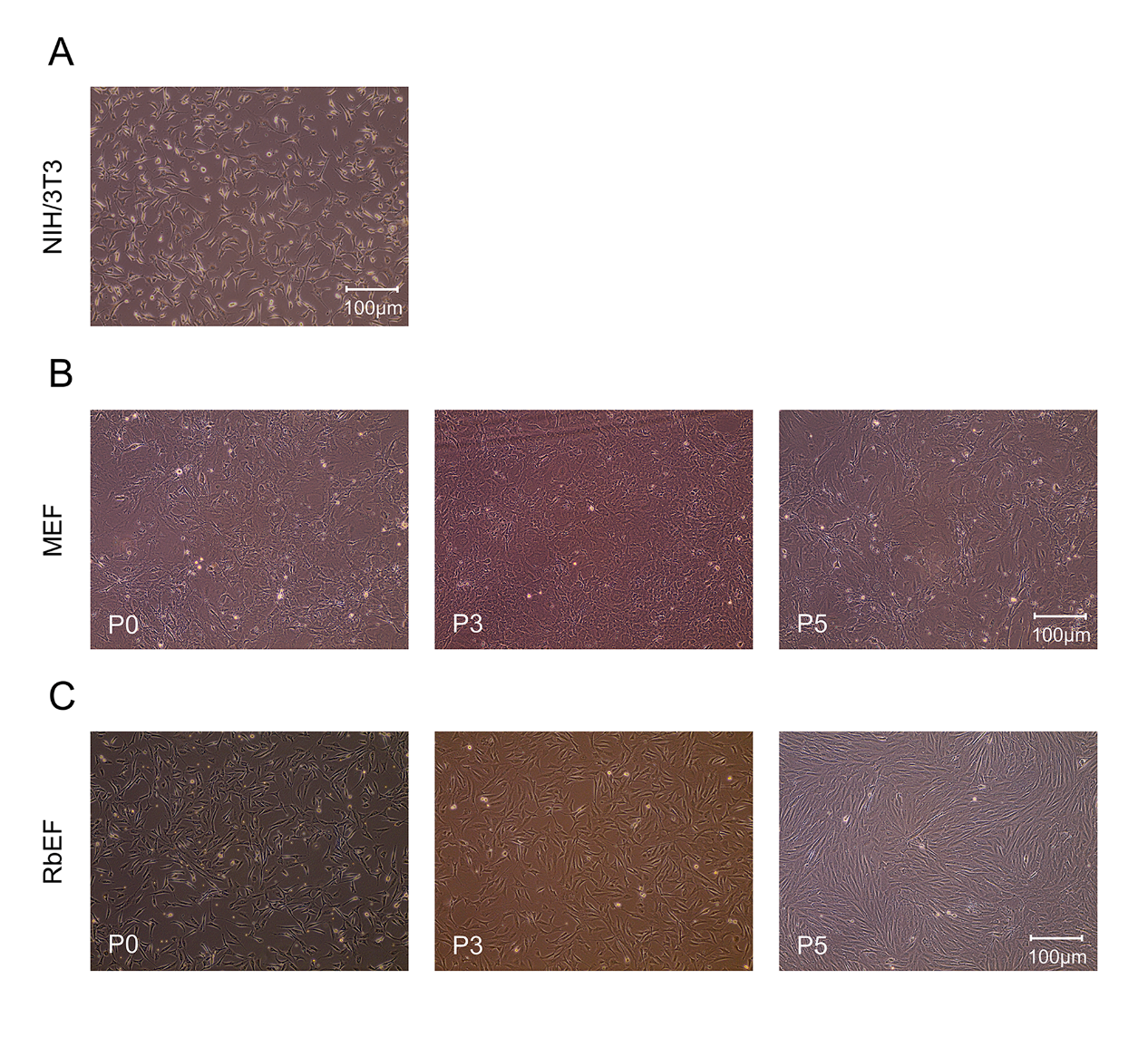


**Fig. S1** Morphological manifestation of NIH/3T3 cells (A), MEFs (B), and RbEFs (C) during culture.


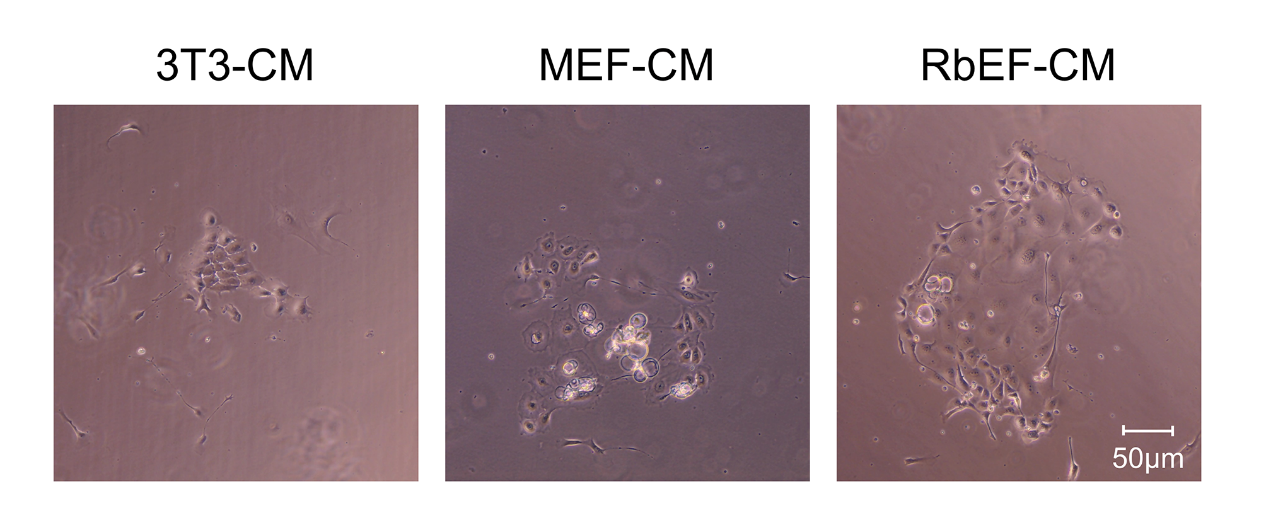


**Fig. S2** Using TrypLE Select for 1-2 minutes to remove extra-clonal spindle cells, and continue to culture mouse ABSCs using 3T3-CM, MEF-CM, or RbEF-CM containing Y-27632.
